# Developing a non-invasive diagnostic model for pediatric Crohn’s disease using RNA-seq analysis

**DOI:** 10.3389/fgene.2023.1142326

**Published:** 2023-03-01

**Authors:** Bin He, Fang Wang, Junhua Shu, Ying Cheng, Xiaoqing Zhou, Tao Huang

**Affiliations:** ^1^ Department of Pediatrics, Fenghua District People’s Hospital of Ningbo, Ningbo, China; ^2^ Department of Pediatrics, Maternal and Child Health Hospital of Hubei Province, Tongji Medical College, Huazhong University of Science and Technology, Wuhan, China

**Keywords:** pediatric Crohn’s disease, inflammatory bowel disease (IBD), RNA-seq, diagnostic model, reproducibility

## Abstract

**Introduction:** Pediatric Crohn’s disease is a chronic inflammatory condition that affects the digestive system in children and adolescents. It is characterized by symptoms such as abdominal pain, diarrhea, weight loss, and malnutrition, and can also cause complications like growth delays and delayed puberty. However, diagnosing pediatric Crohn’s disease can be difficult, especially when it comes to non-invasive methods.

**Methods:** In this study, we developed a diagnostic model using RNA-seq to analyze gene expression in ileal biopsy samples from children with Crohn’s disease and non-pediatric Crohn’s controls.

**Results:** Our results showed that pediatric Crohn’s disease is associated with altered expression of genes involved in immune response, inflammation, and tissue repair. We validated our findings using two independent datasets from the Gene Expression Omnibus (GEO) database, as well as through one prospective independent dataset, and found that our model had a high accuracy rate.

**Discussion:** These findings suggest the possibility of non-invasive diagnosis for pediatric Crohn’s disease and may inform the development of targeted therapies for this condition.

## Introduction

Pediatric Crohn’s disease is a chronic inflammatory condition that affects the digestive system, specifically the small intestine and colon. It is a type of inflammatory bowel disease (IBD) that affects the digestive system in children and adolescents, and it is often diagnosed between the ages of 15 and 25. Symptoms of pediatric Crohn’s disease may include abdominal pain, diarrhea, weight loss, and malnutrition. The disease can also cause complications such as growth delays and delayed puberty in children (Diefenbach and Breuer, 2006). Treatment for pediatric Crohn’s disease may involve medications to reduce inflammation, as well as dietary changes and surgery in some cases. It is important for children with Crohn’s disease to receive regular medical care and follow their treatment plan to manage the disease and prevent complications. (Abraham et al., 2012) Medications used to treat pediatric Crohn’s disease include anti-inflammatory drugs, immune system suppressors, and biologic therapies. Anti-inflammatory drugs, such as corticosteroids, can help reduce inflammation in the digestive tract. Immune system suppressors, such as azathioprine and 6-mercaptopurine, can help prevent the immune system from attacking the digestive tract. Biologic therapies, such as infliximab, work by targeting specific proteins involved in the inflammatory process. The diagnosis of pediatric Crohn’s disease is based on a combination of medical history, physical examination, and test results (Hommel et al., 2013).

To diagnose pediatric Crohn’s disease, a healthcare provider may ask about the child’s symptoms and medical history, including any family history of inflammatory bowel disease. The provider may also perform a physical examination, including a rectal exam, to check for signs of inflammation (de Bie et al., 2013). The accuracy of the diagnosis of pediatric Crohn’s disease can vary depending on the symptoms and test results. In general, the diagnosis of Crohn’s disease is made based on a combination of medical history, physical examination, and test results. Diagnostic tests that may be used to confirm the diagnosis of pediatric Crohn’s disease include blood tests, stool sample analysis, endoscopy, imaging tests, and biopsy. These tests can help identify the presence of inflammation and other signs of Crohn’s disease. However, it is important to note that the accuracy of the diagnosis can sometimes be limited by the variability of the disease and the fact that the symptoms and test results of Crohn’s disease can be similar to those of other conditions. In some cases, a definitive diagnosis of Crohn’s disease may not be possible until the child has had symptoms for a longer period of time and more tests have been performed. Overall, the accuracy of the diagnosis of pediatric Crohn’s disease can be high, but it is important to work closely with a healthcare provider to ensure that the diagnosis is as accurate as possible (Gupta et al., 2008).

In the context of pediatric Crohn’s disease, RNA-seq can be used to identify changes in gene expression that are specific to the disease. For example, studies have shown that pediatric Crohn’s disease is characterized by altered expression of genes involved in immune response, inflammation, and tissue repair. By analyzing the transcriptome of intestinal tissue samples from children with Crohn’s disease, it may be possible to identify specific gene expression patterns that are associated with the disease.

## Methods

### Multi-RNA-seq dataset

To compile the gene expression dataset for this study, we used paired-end RNA-seq data from 304 ileal biopsy samples (GSE101794). These samples included both pediatric Crohn’s patient samples and non-pediatric Crohn’s controls. To further validate our findings, we also accessed two independent datasets from the Gene Expression Omnibus (GEO) database, which is maintained by the National Center for Biotechnology Information (NCBI). The datasets, GSE57945 and GSE93624, comprised 322 and 245 ileal biopsy samples, respectively, and were used to validate our findings. The GEO database can be accessed at https://www.ncbi.nlm.nih.gov/geo/. In addition to the ileal biopsy samples, we also collected blood samples from 13 patients, including 6 pediatric Crohn’s patients and 7 non-pediatric Crohn’s controls. More details and and quantify of expression levels of specific transcripts can be found in the Supplementary Tables S1,S2.

### RNA-seq read mapping

To quantify gene expression levels in the RNA-seq data, we used the nf-core/rnaseq pipeline. This pipeline is an open-source, community-driven pipeline for the analysis of RNA-seq data and is designed to be both reproducible and scalable (Lataretu and Hölzer, 2020). It includes a range of quality control checks, mapping of reads to a reference genome, and quantification of gene expression levels using count-based methods such as featureCounts. The pipeline also includes options for downstream differential expression analysis and visualization of results. To run the pipeline, we first processed the raw RNA-seq data to remove low-quality reads and adaptors using Trimmomatic (Bolger et al., 2014). We then mapped the cleaned reads to the reference genome using Hisat2 and quantified gene expression levels using featureCounts. Finally, we used the nf-core/rnaseq pipeline to perform differential expression analysis and generate a range of visualizations to aid in the interpretation of the results.

### Statistical analysis

In this study, we utilized the R programming language (version 4.2.2) for statistical analysis. To determine significant gene expression differences between pediatric Crohn’s patients and non-pediatric Crohn’s controls, we employed T-tests using the t. test function in the stats package. We also employed principal component analysis (PCA) to visualize the data using the prcomp function in the stats package. In addition, we used an enhanced volcano plot to visualize the results, which is a modified version of the traditional volcano plot that includes additional information such as fold change and *p*-value on the plot. We used the EnhancedVolcano package in R to generate the plots. To identify the most relevant gene set for distinguishing between the two groups, we utilized the forward search function in the MetaIntegrator R package.

## Results

### Gene expression profiles differentiate pediatric Crohn’s disease from non-pediatric Crohn’s disease and identify differentially expressed genes

**FIGURE 1:**
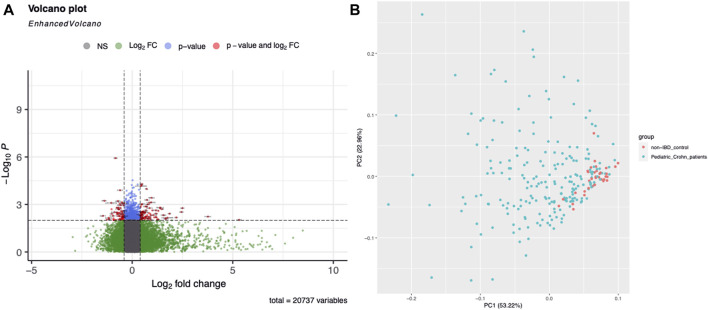
Differential gene expression in Pediatric Crohn’s Disease and controls. **(A)** Enhanced volcano plot showing the log2 fold change and -log10 *p*-value for each gene in the dataset. Genes with a significant difference in expression (*p* < 0.05) are highlighted in red, with those showing an increased expression in Pediatric Crohn’s Disease in blue and decreased expression in green. **(B)** Principal component analysis (PCA) plot illustrating the separation of Pediatric Crohn’s Disease and control samples based on gene expression levels. Each point represents a sample, with Pediatric Crohn’s Disease samples shown in red and controls in blue. The first two principal components (PC1 and PC2) are plotted, representing the majority of the variation in the data.

Gene expression profiling was used to identify differentially expressed genes in 304 ileal biopsy samples from pediatric and non-pediatric Crohn’s disease patients and normal controls. A total of 68 genes were found to be significantly upregulated or downregulated in pediatric Crohn’s disease (*p* < 0.001), and their distribution and fold change values were visualized using a volcano plot (Figure 1A). Principal component analysis (PCA, Figure 1B) showed that there was no significant separation between the pediatric Crohn’s disease and control group, but there was a trend towards separation, suggesting that these differentially expressed genes may be useful for differentiating pediatric from non-pediatric Crohn’s disease and identifying potential biomarkers for this condition (Supplementary Table S3).

### Machine learning-based gene selection and diagnostic model construction

**FIGURE 2:**
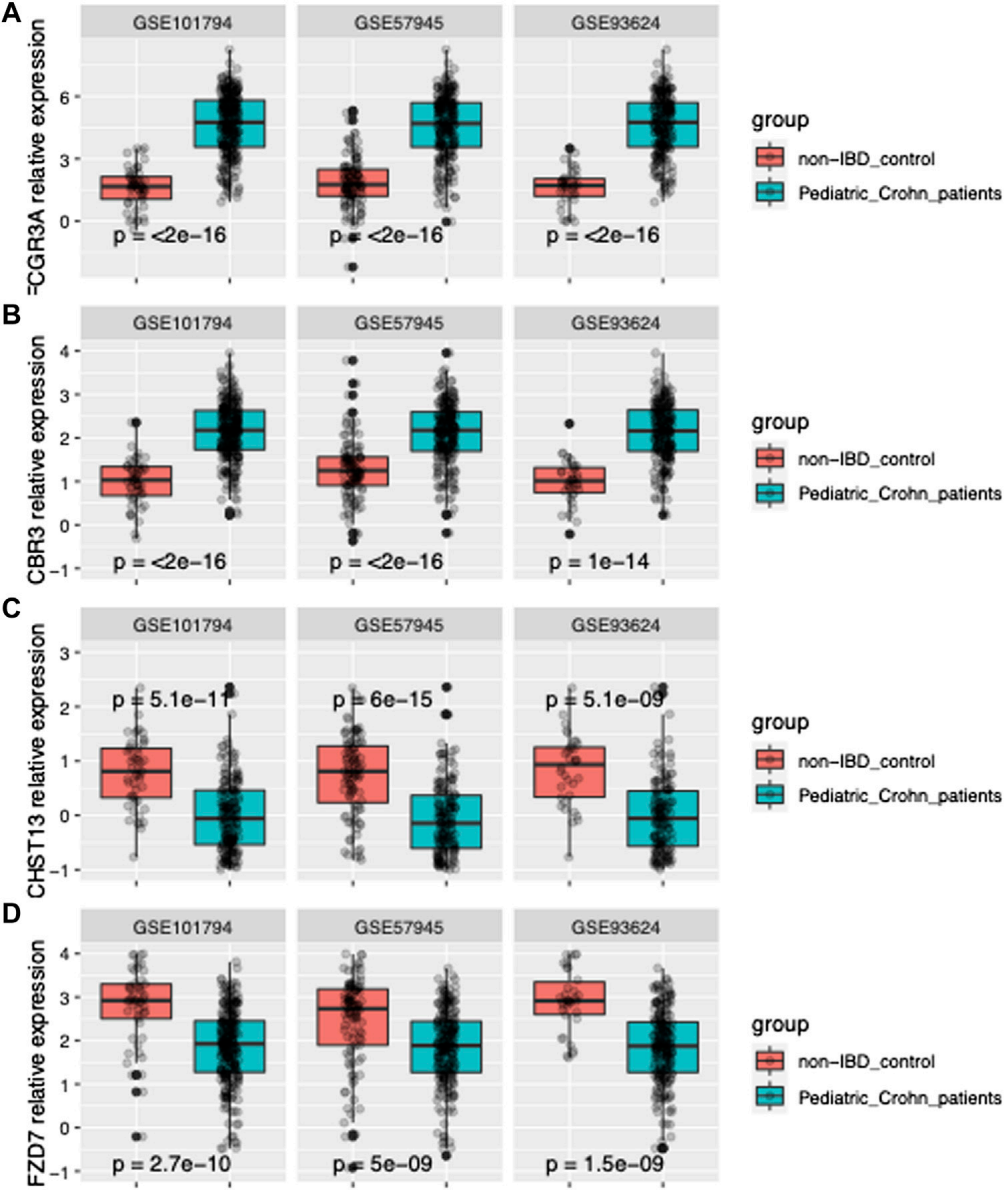
Gene expression of FCGR3A, CBR3, CHST13, and FZD7 in three datasets. **(A)** Boxplot of FCGR3A gene expression in GSE101794, GSE57945 and GSE93624 datasets, representing the pediatric Crohn’s Disease (PD) and control samples. The center line of the box represents the median expression level, the top and bottom edges of the box represent the 75th and 25th percentiles, respectively, and the whiskers extend to the most extreme data points within 1.5 times the interquartile range. The red dots represent outliers. **(B)** Boxplot of CBR3 gene expression in GSE101794, GSE57945 and GSE93624 datasets. **(C)** Boxplot of CHST13 gene expression in GSE101794, GSE57945 and GSE93624 datasets. **(D)** Boxplot of FZD7 gene expression in GSE101794, GSE57945 and GSE93624 datasets.

In order to further improve the diagnostic power of our model, we employed machine learning techniques using the forward search function in MetaIntegrator to identify a smaller set of highly informative genes. Through this process, we identified four differentially expressed genes that were most informative for differentiating pediatric Crohn’s disease from normal/non-pediatric Crohn’s disease (shown in Figure 2A–D). These genes included two upregulated genes (FCGR3A and CBR3) and two downregulated genes (CHST13 and FZD7).

**FIGURE 3:**
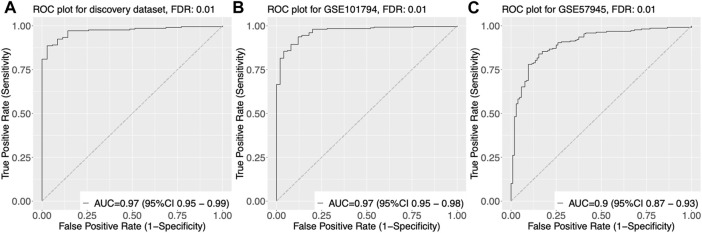
Receiver Operating Characteristic (ROC) curves of gene expression-based classification of Pediatric Crohn’s Disease in three datasets. **(A)** ROC curve of GSE101794 dataset, showing the relationship between the true positive rate (sensitivity) and false positive rate (1-specificity) for different threshold values of gene expression levels for Pediatric Crohn’s Disease classification. The area under the curve (AUC) is indicated. **(B)** ROC curve of GSE57945 dataset, showing the relationship between the true positive rate (sensitivity) and false positive rate (1-specificity) for different threshold values of gene expression levels for Pediatric Crohn’s Disease classification. The area under the curve (AUC) is indicated. **(C)** ROC curve of GSE93624 dataset, showing the relationship between the true positive rate (sensitivity) and false positive rate (1-specificity) for different threshold values of gene expression levels for Pediatric Crohn’s Disease classification. The area under the curve (AUC) is indicated.

We trained and tested various classifiers using these genes as input features and selected the classifier that achieved the highest performance in terms of accuracy, sensitivity, and specificity through cross-validation. Using this optimized classifier, we constructed a diagnostic model that was able to accurately classify samples as pediatric Crohn’s disease or normal/non-pediatric Crohn’s disease, as shown by the high AUC of 0.97 on the ROC curve in Figure 3A.

### Validation of gene signature using public datasets and independent validation set

**FIGURE 4:**
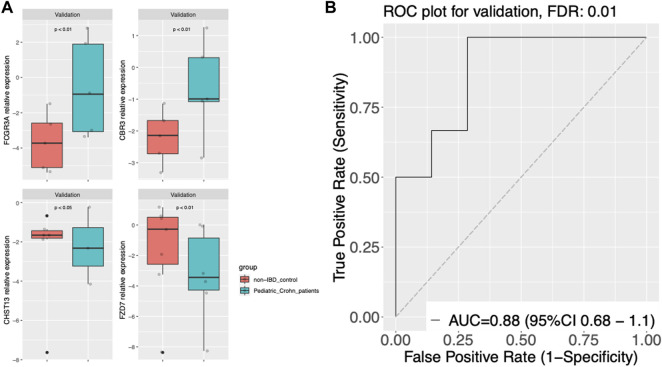
Gene expression and classier performance in an prospective independent validation dataset. **(A)** Boxplots of gene expression for FCGR3A, CBR3, CHST13, and FZD7 in the prospective independent validation set. The center line of the box represents the median expression level, the top and bottom edges of the box represent the 75th and 25th percentiles, respectively, and the whiskers extend to the most extreme data points within 1.5 times the interquartile range. The red dots represent outliers. The boxplots are split into pediatric Crohn’s Disease (PD) and control groups. **(B)** Receiver Operating Characteristic (ROC) curve of gene expression-based classification of Pediatric Crohn’s Disease in the prospective independent validation set. The curve shows the relationship between the true positive rate (sensitivity) and false positive rate (1-specificity) for different threshold values of gene expression levels for Pediatric Crohn’s Disease classification. The area under the curve (AUC) is indicated.

To validate the diagnostic power of our gene signature, we used two independent approaches. First, we tested the performance of our model on publicly available datasets. We obtained gene expression data from GSE57945 and GSE93624 datasets, and applied our diagnostic model to classify samples as pediatric Crohn’s disease or normal/non-pediatric Crohn’s disease. The results showed that our model achieved high accuracy, as demonstrated by the AUC of 0.97 and 0.9 on the ROC curve for these two datasets, respectively (Figure 3A–C). This indicates that our model was able to generalize to independent samples. Second, we also tested the performance of our model on an independent validation set that was not used in the training or testing of the model. This validation set consisted of 6 samples from pediatric Crohn’s disease patients and 7 samples from normal controls. The results showed that our model was able to accurately classify samples as pediatric Crohn’s disease or normal/non-pediatric Crohn’s disease with high accuracy (AUC = 0.88, Figure 4). Supplementary Table S4 provides ROC curve analysis results for four data sets, including information on accuracy, sensitivity, and specificity. Overall, these results demonstrate the robustness and generalizability of our gene signature for differentiating pediatric Crohn’s disease from normal/non-pediatric Crohn’s disease. The high accuracy of our model on both public datasets and independent validation set suggests that our gene signature has the potential to be used as a diagnostic tool for pediatric Crohn’s disease.

## Discussion

Our study identified four differentially expressed genes that were most informative for differentiating pediatric Crohn’s disease from normal/non-pediatric Crohn’s disease. We utilized GSE101794 as the discovery set to identify signature genes and establish a model. Through DEseq2 method, we identified *p*-values, Fold change, and FDR values for each gene, resulting in volcano plot and PCA analysis. The PCA analysis revealed that the case and control groups were not well separated. Therefore, we employed MetaIntegrator’s machine learning algorithm and identified four differentially expressed genes. The diagnostic model constructed using these four differentially expressed genes demonstrated excellent diagnostic performance on three publicly available datasets. We subsequently validated the model in an prospective independent validation dataset. These genes included two upregulated genes (FCGR3A and CBR3) and two downregulated genes (CHST13 and FZD7). FCGR3A is a gene encoding the Fc fragment of IgG, receptor IIIa, which is a surface receptor expressed on various immune cells, including monocytes, macrophages, and neutrophils. Previous studies have shown that FCGR3A is involved in immune responses and inflammation, and its expression is upregulated in various autoimmune and inflammatory diseases, such as rheumatoid arthritis and lupus erythematosus (Breunis et al., 2009). Our findings suggest that FCGR3A may play a role in the pathogenesis of pediatric Crohn’s disease, potentially through its involvement in immune responses and inflammation. CBR3 is a gene encoding carbonyl reductase 3, which is a member of the aldo-keto reductase family and involved in the metabolism of various endogenous and exogenous compounds. Previous studies have shown that CBR3 is involved in the metabolism of xenobiotics, such as drugs and environmental toxins, and its expression is upregulated in various cancer types (Blanco et al., 2008). Our findings suggest that CBR3 may be involved in the pathogenesis of pediatric Crohn’s disease. CHST13 is a gene encoding carbohydrate sulfotransferase 13, which is a member of the sulfotransferase family and involved in the sulfation of various carbohydrates. Previous studies have shown that CHST13 is involved in the synthesis of proteoglycans and glycosaminoglycans, which are important components of the extracellular matrix and involved in tissue development and repair. Our findings suggest that CHST13 may be involved in the pathogenesis of pediatric Crohn’s disease, potentially through its role in extracellular matrix homeostasis and tissue repair. FZD7 is a gene encoding Frizzled 7, which is a member of the Frizzled family and a receptor for Wnt signaling. Previous studies have shown that FZD7 is involved in various developmental and physiological processes, such as cell proliferation, differentiation, and migration (Guo et al., 2019). Our findings suggest that FZD7 may be involved in the pathogenesis of pediatric Crohn’s disease, potentially through its role in the regulation of these processes.

As we all known, RNA-seq can not only be used to identify differentially expressed encoding genes but also be used to explore non-coding genes in patient samples. There is a growing body of evidence suggesting that long non-coding RNAs (lncRNAs) may play a role in the development of Crohn’s disease and colorectal cancer (Yarani et al., 2018; Chen and Shen, 2020). For example, lncRNA THOR expression was significantly increased in colorectal cancer tissue compared to normal prostate tissue associating with poor patient outcomes (Chu et al., 2020). Other studies have also shown that lncRNA THOR is overexpressed in other types of cancer, including endometrial cancer and ovarian cancer (Ge et al., 2020; Zhang et al., 2022). While some studies have identified lncRNAs that are differentially expressed in pediatric Crohn’s disease patients and have suggested their potential as therapeutic targets or diagnostic biomarkers, more research is needed to fully understand their role in the development and progression of this condition (Haberman et al., 2018; Lucafò et al., 2019). Additionally, further studies are needed to determine the relationship between lncRNA expression and patient outcomes in pediatric Crohn’s disease, as this information could potentially be used to guide treatment decisions and improve patient outcomes. Therefore, we will continue to investigate the profiles of lncRNAs in pediatric Crohn’s disease in our future research.

Overall, our findings provide new insights into the molecular mechanisms underlying pediatric Crohn’s disease and identify potential therapeutic targets for this disease. Further studies are needed to confirm the roles of these genes in pediatric Crohn’s disease and to investigate their therapeutic potential.

## Data Availability

The datasets presented in this study can be found in online repositories. The names of the repository/repositories and accession number(s) can be found in the article/Supplementary Material.
