# Supplementary material for: Developing a non-invasive diagnostic model for pediatric Crohn’s disease using RNA-seq analysis
Source: Front Genet. 2023 Mar 1;14:1142326. doi: 10.3389/fgene.2023.1142326 (PMC10014721; doi:10.3389/fgene.2023.1142326)
Supplement: Supplementary file 1 [file DataSheet1.docx]

**Developing a Non-Invasive Diagnostic Model for Pediatric Crohn's Disease Using RNA-seq Analysis**

Supplementary Table S1. Clinical Information of Patients with Pediatric Crohn's Disease and Control Group.

| **Patient ID** | **Age** | **Gender** | **Ethnicity** | **Diagnosis** |
| --- | --- | --- | --- | --- |
| 1 | 12 | Male | Asian | Pediatric Crohn's |
| 2 | 14 | Female | Asian | PediatricCrohn's |
| 3 | 15 | Male | Asian | PediatricCrohn's |
| 4 | 8 | Female | Asian | PediatricCrohn's |
| 5 | 10 | Male | Asian | PediatricCrohn's |
| 6 | 16 | Female | Asian | PediatricCrohn's |
| 7 | 18 | Male | Asian | Non-PediatricCrohn's |
| 8 | 17 | Female | Asian | Non-PediatricCrohn's |
| 9 | 16 | Male | Asian | Non-PediatricCrohn's |
| 10 | 15 | Female | Asian | Non-PediatricCrohn's |
| 11 | 14 | Male | Asian | Non-PediatricCrohn's |
| 12 | 13 | Female | Asian | Non-PediatricCrohn's |
| 13 | 12 | Male | Asian | Non-PediatricCrohn's |

Supplementary Table S2. The primer sequences for FCGR3A, CBR3, CHST13, and FZD7

**RT-PCR assay**

To quantify the expression levels of specific transcripts in the samples, we performed RT-PCR assays. The cDNA was then synthesized from the total RNA using the High Capacity Reverse

Transcription kit (ABI, Cat# 4368814) according to the manufacturer’s protocol. The qPCR methodology and primers were designed and used according to the protocols. (QIAGEN,

cat. No. 205143)

| **Gene** | **Forward Primer Sequence(5'-3')** | **Reverse Primer Sequence(5'-3')** |
| --- | --- | --- |
| FCGR3A | GGTGACTTGTCCACTCCAGTGT | ACCATTGAGGCTCCAGGAACAC |
| CBR3 | ATGTCATGGTTCTCCCCAAA | CTGTGCCCTCTGTTCCAACT |
| CHST13 | TAATAGTAATCAATTACGGG | CCTCTACAAATGTGGTATGGC |
| FZD7 | CTACCCTACCGCGCCCTAC | CATGAAGTAGCAGCCCGACA |
| ACTB | TGGTGAGCAGAGGAGATGATG | CAGGGCAGAGGGACTTACTT |

Supplementary Table S3. DEseq2 Results for the Top 100 Genes in the Discovery Set - P-values and Fold Changes

| gene | avg_1 | avg_2 | log2FC | pvals |
| --- | --- | --- | --- | --- |
| SCIN | 5.76170824 | 4.93194666 | -0.8297616 | 1.18E-06 |
| POP4 | 3.07437378 | 3.09375084 | 0.01937706 | 2.96E-05 |
| ASS1 | 7.32658528 | 7.80839262 | 0.48180735 | 5.14E-05 |
| RP11-85B7.2 | -6.8851705 | -6.6155437 | 0.2696268 | 5.46E-05 |
| TDRP | 1.29926685 | 1.28096142 | -0.0183054 | 5.92E-05 |
| STAT2 | 4.44663258 | 4.91073509 | 0.46410251 | 6.03E-05 |
| LOC100996634 | -2.681433 | -2.2417943 | 0.43963872 | 6.71E-05 |
| RPL36A-HNRNPH2 | 5.13333711 | 5.21484448 | 0.08150737 | 8.34E-05 |
| ELAC2 | 3.3676866 | 3.38906722 | 0.02138061 | 9.03E-05 |
| PRL | -3.3532565 | -2.6361861 | 0.71707043 | 0.00010511 |
| RGAG4 | -0.3821575 | -0.0461496 | 0.3360079 | 0.000117 |
| CYP2U1 | 2.37311908 | 1.77037376 | -0.6027453 | 0.00012647 |
| LOC101929512 | -2.8790715 | -3.1570175 | -0.277946 | 0.00013547 |
| USP33 | 3.62112753 | 3.60698595 | -0.0141416 | 0.00014645 |
| SLAIN2 | 2.81300203 | 2.773363 | -0.039639 | 0.00017941 |
| FOXD4L1 | -3.3970203 | -3.464631 | -0.0676106 | 0.00019687 |
| BRSK2 | -2.5511817 | -2.6974181 | -0.1462364 | 0.00020041 |
| LOC101927188 | -4.7574209 | -4.7453697 | 0.01205112 | 0.00020619 |
| ZNF496 | 2.02129067 | 2.13647448 | 0.11518381 | 0.00022775 |
| PRDM11 | -0.8068164 | -0.9930358 | -0.1862194 | 0.00025262 |
| SAP18 | 5.01885887 | 4.98640645 | -0.0324524 | 0.00025741 |
| ACHE | 3.28898517 | 3.0210085 | -0.2679767 | 0.00026828 |
| SBF2 | 1.6563248 | 1.8445733 | 0.1882485 | 0.00029293 |
| TOMM22 | 4.9294014 | 4.96782475 | 0.03842335 | 0.00029995 |
| PDE1C | -0.5859799 | -0.4710978 | 0.11488213 | 0.00030002 |
| NCBP1 | 2.93918699 | 2.91814924 | -0.0210378 | 0.00033718 |
| LOC101928816 | -1.7183623 | -1.7983013 | -0.0799389 | 0.00035761 |
| EEF1G | 9.68296529 | 9.60234491 | -0.0806204 | 0.00036889 |
| FOXK1 | 1.49392542 | 1.40143419 | -0.0924912 | 0.0003721 |
| RN7SL2 | 10.1556767 | 11.1185658 | 0.9628891 | 0.00038115 |
| LRRC37A4P | 1.80766596 | 1.50080249 | -0.3068635 | 0.00042489 |
| PPIA | 9.26796435 | 9.41800569 | 0.15004134 | 0.00044528 |
| C11orf63 | -0.1942328 | -0.5029217 | -0.308689 | 0.00048141 |
| CALM3 | 7.10974366 | 7.12988356 | 0.02013991 | 0.00050355 |
| RPEL1 | -5.326137 | -4.5873556 | 0.73878144 | 0.00051667 |
| LRRC2 | -1.683807 | -1.8392362 | -0.1554292 | 0.00054493 |
| CBLN2 | -0.4811552 | -0.8587722 | -0.377617 | 0.00056069 |
| APOA1 | 11.0873051 | 9.70369379 | -1.3836114 | 0.00058573 |
| MAP3K4 | 2.35505678 | 2.1595606 | -0.1954962 | 0.00060097 |
| AKAP6 | -1.1079311 | -1.818669 | -0.7107379 | 0.00061168 |
| CLSTN1 | 4.85659922 | 4.89060264 | 0.03400343 | 0.00062148 |
| ARHGAP5 | 3.04754588 | 2.78965283 | -0.257893 | 0.00066285 |
| NR1D2 | 3.00360965 | 2.71619257 | -0.2874171 | 0.00067711 |
| ZNF491 | -0.3219999 | -0.4838224 | -0.1618225 | 0.00067762 |
| SP3 | 3.66491635 | 3.60175832 | -0.063158 | 0.00070524 |
| FLJ12825 | -3.2522428 | -3.7700731 | -0.5178303 | 0.00072962 |
| LOC285762 | -6.5566184 | -5.615735 | 0.94088338 | 0.00073431 |
| CRYM-AS1 | -3.4623338 | -3.7121885 | -0.2498548 | 0.00073939 |
| DNM1L | 3.13591182 | 3.2377459 | 0.10183409 | 0.0007602 |
| ZNF652 | 1.78657256 | 1.55944123 | -0.2271313 | 0.00079025 |
| EXOC6 | 2.20590332 | 2.38478761 | 0.17888429 | 0.00079028 |
| LOC101929733 | 1.60342882 | 0.55830121 | -1.0451276 | 0.00079159 |
| GRIN2D | -2.4440143 | -1.5873724 | 0.85664187 | 0.00079533 |
| CLPP | 4.79001639 | 4.85009475 | 0.06007836 | 0.0008028 |
| MRPS23 | 3.33622156 | 3.54295845 | 0.20673689 | 0.00082343 |
| POPDC2 | 0.72263771 | 0.53753415 | -0.1851036 | 0.00085593 |
| CD96 | 3.03900812 | 2.5554282 | -0.4835799 | 0.00085818 |
| FAM91A1 | 3.14303797 | 3.20990315 | 0.06686519 | 0.00086021 |
| YY1AP1 | 4.40444095 | 4.35324197 | -0.051199 | 0.00087258 |
| PHACTR1 | 2.00138273 | 1.59899186 | -0.4023909 | 0.00088591 |
| IDH3A | 4.78875249 | 4.82044395 | 0.03169146 | 0.00091147 |
| CDC14B | 2.39027758 | 2.2221387 | -0.1681389 | 0.00093188 |
| SUGCT | 2.13849396 | 1.51483709 | -0.6236569 | 0.00093488 |
| LOC102723714 | 3.05099705 | 3.21700683 | 0.16600979 | 0.00094783 |
| JAKMIP1 | -1.2244848 | -0.731489 | 0.49299577 | 0.00094973 |
| C7orf57 | -4.8166678 | -5.0854376 | -0.2687698 | 0.00095282 |
| GBP6 | -4.1614526 | -3.383167 | 0.77828562 | 0.00099057 |
| AP3B1 | 3.5326682 | 3.65885138 | 0.12618318 | 0.00102856 |
| SEMA4C | 2.36424692 | 2.68377322 | 0.3195263 | 0.00103412 |
| ERICH2 | -2.9067709 | -2.4454321 | 0.46133873 | 0.0010402 |
| HHAT | 0.51358719 | 0.46268619 | -0.050901 | 0.00105994 |
| ASTN2 | 0.79005482 | 0.42465743 | -0.3653974 | 0.00106231 |
| SUPT6H | 3.80005951 | 3.72756204 | -0.0724975 | 0.00106308 |
| UNC5C | -0.1853 | -0.1271382 | 0.05816172 | 0.00108452 |
| TADA1 | 2.38875841 | 2.21915457 | -0.1696038 | 0.00109017 |
| THADA | 2.2353727 | 2.37284979 | 0.13747709 | 0.00109906 |
| NEFH | -0.9045919 | -0.852179 | 0.05241296 | 0.00110477 |
| ANKRD55 | -0.6929497 | -1.2603272 | -0.5673774 | 0.00111557 |
| LIN7A | 1.12195604 | 0.69670223 | -0.4252538 | 0.00112701 |
| TBCA | 6.31706599 | 6.32986786 | 0.01280188 | 0.00115226 |
| MTERFD2 | 3.82587622 | 3.58100304 | -0.2448732 | 0.00116184 |
| TRAPPC12 | 3.49098489 | 3.49128895 | 0.00030407 | 0.00118695 |
| IQGAP1 | 4.70562 | 4.80976806 | 0.10414806 | 0.00122157 |
| TMOD3 | 3.75944444 | 3.79078842 | 0.03134398 | 0.00123255 |
| CLASP2 | 2.10877874 | 2.0307609 | -0.0780178 | 0.00124228 |
| SLC35B1 | 4.86470723 | 4.78925426 | -0.075453 | 0.00126421 |
| VCL | 3.80063678 | 4.08244837 | 0.28181159 | 0.00131019 |
| ESAM | 2.56662899 | 3.23454895 | 0.66791996 | 0.00133567 |
| SPAG9 | 3.51393254 | 3.47956339 | -0.0343691 | 0.00134062 |
| GABPA | 3.03728222 | 2.8810784 | -0.1562038 | 0.00134862 |
| LILRB1 | 2.76883377 | 3.48606166 | 0.71722788 | 0.00135578 |
| NT5DC1 | 3.97222052 | 3.64542102 | -0.3267995 | 0.00136409 |
| LOC101927608 | -3.7253135 | -3.4122714 | 0.31304213 | 0.00137405 |
| ALAS1 | 4.43252531 | 4.86052001 | 0.4279947 | 0.00141493 |
| DCAF10 | 2.17771453 | 2.12077099 | -0.0569435 | 0.00146054 |
| CA13 | 2.71827073 | 2.46285977 | -0.255411 | 0.00146449 |
| LINC00998 | 4.09313545 | 4.35113666 | 0.25800121 | 0.0014829 |
| ACLY | 4.62387053 | 4.45176997 | -0.1721006 | 0.00148673 |
| EARS2 | 1.70399898 | 1.90541073 | 0.20141175 | 0.00150179 |

Supplementary Table S4. ROC Curve Analysis Results for Four Data Sets.

| Dataset ID | sens | spec | PPV | NPV | acc |
| --- | --- | --- | --- | --- | --- |
| GSE101794 | 0.909 | 0.88 | 0.975 | 0.657 | 0.905 |
| GSE57945 | 0.775 | 0.904 | 0.944 | 0.657 | 0.817 |
| GSE93624 | 0.924 | 0.886 | 0.98 | 0.66 | 0.918 |
| Independent validation | 0.667 | 0.714 | 0.667 | 0.714 | 0.692 |

sens = Sensitivity

spec = Specificity

PPV = Positive Predictive Value

NPV = Negative Predictive Value

acc = Accuracy
